# Clinical Decision-Making in Chronic Spine Pain: Dilemma of Image-Based Diagnosis of Degenerative Spine and Generation Mechanisms for Nociceptive, Radicular, and Referred Pain

**DOI:** 10.1155/2018/8793843

**Published:** 2018-12-17

**Authors:** Haytham Eloqayli

**Affiliations:** ^1^Department of Neurosurgery, Faculty of Medicine, Jordan University of Science and Technology (JUST), Irbid 22110, Jordan; ^2^Emirate Specialty Hospital, Dubai Healthcare City, P.O. Box 66566, Dubai, UAE

## Abstract

**Background:**

Spine-related pain is a complex heterogeneous condition. Excessive reliance on radiological imaging might lead to overdiagnosis of incidental asymptomatic spinal changes and unnecessary surgery. Approaches to the clinical management of spine pain should (1) identify pain generators, types, patterns, and mechanisms; (2) confirm clinical suspension with a diagnostic injection; and (3) ensure that treatment is aimed at controlling pain and improving patient function rather than image-based surgical success.

**Method:**

This case series (7 cases) discusses commonly seen clinical presentation of spine pain analytically, with illustrations of possible pain generators, mechanisms, pathways, and pain types. Each case discusses pain types and location (axial nociceptive, referred, and radicular neuropathic), generators (degenerated disc, herniated disc, facet joint, and sacroiliac joint), pathways (sinuvertebral ventral ramus and medial and lateral branches dorsal ramus), and radiculopathy versus radicular pain, elaborating on coccydynia and cervicogenic headaches, epimere versus hypomere muscle embryology, function, innervation, and role in spine-related pain.

**Results:**

Multiple pain generators might coexist in the same patient causing mixed pain types and referral patterns with multiple mechanisms and pathways. History review, physical examination, and diagnostic injections are the mainstays of diagnosis.

**Conclusions:**

Image-detected spondylosis might be an asymptomatic process. Clinical presentation is related to stenosis or pain. The mechanism of pain is related to compression, inflammation, or microinstability. Spine pain can be nociceptive axial, neuropathic radicular, and/or referred pain. Although image findings are helpful in radicular neuropathic pain from disc herniation, they are unreliable in nociceptive pain, and correlation with clinical and diagnostic injections is mandatory.

## 1. Introduction

Chronic spine pain is an increasingly prevalent multifactorial heterogeneous pain syndrome often lasting beyond 12 weeks [1]. Considerable controversy still exists over a variety of issues: the correlation between spine degeneration and pain, image-guided versus pain guided treatment, pain types, mechanisms and generators, the weight of predisposing and risk factors, cervicogenic headache, and referral versus radicular pain patterns [2]. A new look at this painful condition mandates that therapeutics should be aimed at pain relief and return to functional life rather than operation success alone, which needs an approach that integrates basic, clinical, and surgical scientific knowledge [3].

The high occurrence of asymptomatic disc degeneration with the absence of significant image difference between controls and spine pain patients [4, 5] emphasizes the importance of two issues. The first is history and physical examination which is being undermined with the evolution of increasingly sophisticated anatomical diagnostics, and the second is the need for new functional noninvasive diagnostics or blood biomarkers that can guide the pain process and response in an objective manner. The increase in image findings-based treatment with the complexity and heterogeneity of spine pain may risk overdoing unnecessary invasive procedures.

More than one pain type (nociceptive, neuropathic, and referred) or pain generator (disc, facet, sacroiliac, and muscle) may coexist in the same patient over the same period. Therefore, an analytical clinical approach combined with deep basic scientific knowledge is needed. The hallmark of axial pain is nociceptive inflammation, whereas radicular pain is a neuropathic type. Radiculopathy and radicular pain are not synonymous and need to be differentiated from referred pain. Cervicalgia needs to be differentiated from a migraine. Pain mediated via ventral rami that could refer to epimere-originating muscles should be differentiated from pain mediated via the dorsal rami and might affect hypomere-originating deep postural spine muscles.

Consequently, anatomical diagnosis (i.e., lumbar disc, spine degeneration) can be misleading in pain patients and may justify unnecessary interventions if not correlated well, analytically.

The first aim of this study was to illustrate the approach to pain management from a functional perspective, with a correlation of clinical picture, pain type, mechanism generators, and image findings, emphasizing that anatomical diagnoses can be asymptomatic and do not always correlate with patient complaints. The second aim was to provide education for students, family doctors general practitioners, residents, chiropractors, physiotherapists, and paramedics. This is done by focusing on the clinical differentiation of nociceptive, referred, and neuropathic spine-related pain, differentiating radiculopathy from radicular pain, and by clinically differentiating between sinuvertebral discogenic medial branch facet joint and lateral branch sacroiliac joint and epimere ventral rami innervated and hypomere dorsal rami innervated muscles.

## 2. Case Series

### 2.1. Case Presentation

#### 2.1.1. Case 1

A 27-year-old female patient presented with recurrent left-sided, lower back and left gluteal pain for 6 months. The lower back pain increased with long periods of sitting or standing, whereas the gluteal pain was most intense when changing positions, particularly when standing from a sitting position. Both types of pain improved partially with walking. One month prior to visiting the clinic, she noticed occasional numbness in the left thigh. She showed a partial temporary response to nonsteroidal anti-inflammatory drugs (NSAIDs). Neurological examination was normal, except for pain on spinal extension and axial rotation, with tenderness over the lower lumbar paraspinal region and left sacroiliac joint (SIJ) region. She underwent a lumbar stress X-ray (LSS-X-ray), sacroiliac joint X-ray, and lumbar MRI which showed moderate left L5/S1 posterolateral disc prolapse. Her pain score was described as 8/10.

The clinical picture of this patient can suggest facet joint pain or sacroiliac joint (SIJ) pain, whereas the MRI findings showed intervertebral disc (IVD) herniation. It is important to note that the image changes for facet and sacroiliac joints do not correlate with the pain experienced by the patients. Consequently, normal images cannot exclude facet or SIJ as pain generators. Therefore, diagnostic injections are the only confirmatory tests. Facet joint pain is mediated with the medial branch of the dorsal rami where each facet joint is innervated with two medial branches: from the level above and the same level (i.e., L3/4 facet is innervated with L2 and L3 medial branches). Hence, a diagnostic medial branch block should involve the two supplying medial branches. The L4 lateral branch, L5 dorsal ramus, and S1,2,3 lateral branches innervate SIJ posteriorly. The presence of disc herniation could be coincidental, as the expected radiculopathy and radicular pain were both absent. Patient underwent diagnostic L3 and L4 medial branch (for the L4/5 facet), L5 dorsal ramus, and S1,2,3 lateral branch blocks. The pain VAS score dropped to 2/10 which confirmed the diagnosis of facet joint and SIJ pain. Selective nerve root block, which was planned if the patient's responses were negative for medial and lateral branches blocks, was canceled since a positive response was obtained. The patient was referred for physiotherapy, with a plan for radiofrequency if symptoms recurred.

#### 2.1.2. Case 2

A 54-year-old male patient presented with a two-day history of severe left-sided, lower back pain which disappeared with the appearance of left-sided lower limb pain felt deep inside the whole lower limb and described by the patient as intense internal pressure (VAS 8/10). On examination, power was 5/5 with no sensory deficit and normal reflexes. The patient received NSAID painkillers and active bed rest was advised. However, on the patient's insistence, a lumbar spine X-ray and MRI were done which showed mild IVD protrusion. The patient was reassured and sent home. A week later the patient presented to the ER with an 18-hour history of heaviness and difficulty in raising the left foot when walking, with numbness along the lateral part of the leg and dorsum of the foot. The pain was moderate (VAS 5/10). No sphincter-related symptoms were observed. According to the patient, the decreased pain caused the delay in presentation, against the instructions on first evaluation. On examination of the foot dorsiflexion was 2/5 (movement on gravity alleviation) with decreased sensation along the left L5 dermatome. There were normal reflexes and sphincters. The patient underwent immediate, new lumbar MRI which showed a large disc sequester with disc migration. His status was fully explained; he was admitted and underwent microdiscectomy L4/5.

The initial back pain lasted for 2 days, mostly caused by stretching and pressure on the weakened annulus fibrosis. This type of pain disappears or decreases once the annulus opens and nucleus pulposus leaks to the spinal or root canal. Initial left lower limb pain is a typical description of painful radiculopathy where mass pressure and inflammatory irritation of the nerve root and dorsal root ganglion cause neuropathic pain. This is different from nociceptive and referred pain types. Dorsal root ganglion hosts the cell bodies of sensory nerves with bidirectional connections (to the periphery and spinal cord). Injury to the nerve root motor fibers causes weakness.

The majority of patients with lumbar disc and radicular pain improve with conservative treatment. Surgical indications are acute or progressive motor weakness, sphincter dysfunction, intractable pain not responding to analgesia, and pain affecting patient daily life, not responding to 6 weeks of conservative treatment in the absence of acute surgical indications. In all cases, there should be a correlation between the clinical picture and MRI findings. In the current case, the patient has left L5 nerve root symptoms and signs (dorsiflexion weakness, normal reflexes, and dermatologic radiculopathy). The expected IVD prolapse occurred on either the left posterior-lateral L4/5 or left extreme lateral L5/S1 disc. Although there is a debate about dermatologic sensory distribution in root-originating symptoms and variations in clinical presentation (i.e., the L4/5 disc causing S1 root symptoms via compressing the root in higher position, groin pain with lower lumbar discs due to paravertebral sympathetic ganglion pathway entering at L1 or L2 nerve), we presented the commonly encountered scenarios.

#### 2.1.3. Case 3

A 70-year-old female patient presented with recurrent lower back pain (LBP) which worsened during the last 6 months. The pain increased with changing position and did not improve with walking. Alternating pain radiated to the gluteal region, thighs, and legs with pain in the left more than the right side (VAS for LBP 9/10, for lower limb pain 3/10). Examination showed only decreased sensations in the L5 dermatomal distribution. The patient underwent a lumbar MRI and stress X-ray which showed L4/5 degenerative spondylolisthesis grade 2. Instrumental fusion and decompression were initially planned, but both the patient and family declined surgical intervention. She was offered a palliative solution of a medial branch block which showed a good response (VAS for LBP decreased from 9 to 2). The patient was referred for physiotherapy, prescribed a lumbar belt and painkillers, and scheduled for radiofrequency on symptom recurrence.

Spondylolisthesis refers to the forward or backward slippage of one vertebra on an adjacent vertebra. Isthmic spondylolysis and spine degeneration are the most common causes. The first involves a defect in pars interarticularis occurring in young athletes, whereas the latter occurs due to degenerative facets in older subjects and, possibly, an increase in sagittal-orientated facet joints. Degenerative spondylolisthesis tends to be asymptomatic and self-limiting. Hence, surgery is reserved for patients with significant pain affecting daily life. Pain generation could arise from mechanical instability and/or nerve compression. Consequently, in the absence of central or lateral canal stenosis or significant symptoms, pain can be approached via minimal interventions, mainly medial and lateral branch blocks and radiofrequency. It should be noted that two diagnostic injections are needed to confirm the pain source. However, in the current case, the aim was palliative pain treatment and we considered a single injection enough.

#### 2.1.4. Case 4

A 48-year-old female patient presented with recurrent neck pain extending to both shoulders, affecting her daily activity and sleep. She responded partially to physiotherapy and painkillers. On examination, there was muscle spasm mainly in the upper trapezius with limitation to neck extension, lateral flexion, and rotation. She underwent a cervical X-ray and MRI which showed two moderate IVD protrusions at C4/5 and C5/6. The patient underwent percutaneous discectomy for both discs under local anesthesia and sedation. Referred pain to upper limbs and shoulders disappeared the same day (VAS dropped from 5 to 1), whereas neck pain decreased significantly (VAS dropped from 8 to 2). For 3-18 days, the patient reported intermittent attacks of neck pain (VAS 6/10) but not referred pain. After 18 days her axial neck pain showed mild improvement (VAS 4/10). She began physiotherapy for strengthening deep muscles and relaxing extrinsic muscles and showed good improvement for 4 months.

The initial pain improvement could be attributed to decreased disc pressure. Referred pain arises from any of the pain generators in the same spinal segment. However, axial neck pain could be single or a combination of discogenic, facet joint, and/or myofascial pain. Asymptomatic spine degeneration is common and nonlocalizing for nociceptive pain; therefore, diagnostic injection and discography correlation with patient axial pain and image findings are necessary. Discography is done as part of the percutaneous discectomy, and it requires the patient to be fully awake, which could be difficult to accomplish in cervical procedures. Consequently, discography is a confirmatory test in image-guided percutaneous procedures which form a bridge between conservative and classical surgical interventions. The current patient may need a medial branch block on axial pain recurrence.

#### 2.1.5. Case 5

A 42-year-old female patient presented with neck pain, right brachialgia, right occipital pain, and right-sided pain radiating to the frontal and periorbital region for 8 months. Pain attacks occurred 3-5 times weekly with variation in intensity for each pain component (average VAS 7/10). The patient was on painkillers and treatment for a migraine. Physical examination showed decreased sensation along the right C6 nerve distribution, tender neck muscles with limited neck movement, and trigger points with referred myofascial pain to the occipital, frontal, and periorbital region. Cervical MRI showed multiple small disc protrusions and a moderate disc at right C5/6. The patient underwent surgical treatment for the C5/6 disc which relieved her symptoms including occipital, frontal, and periorbital pain.

The current patient presented with axial, referred, and radicular pain, but the hemisided headache similar to a migraine was different. Cervicogenic headache is pain perceived in the trigeminal region caused by changes in the bony or soft neck structures. Though cervicalgia is reported with lower cervical discs, a debate about the mechanism still exists, and it is occasionally mistaken for a migraine or trigeminal neuralgia. Possible mechanisms and the author's personal scientific opinion are included for completion in the discussion.

#### 2.1.6. Case 6

A 38-year-old female office worker presented with pain at the coccygeal region (tailbone) recurrent for 6 months with short periods of pain relief during vacation. Her pain increased with sitting on a hard object and did not respond to conservative treatment. There was no neurological deficit. X-ray showed type I coccyx (curved slightly forward). She underwent fluoroscopic guided impar ganglion block (sacrococcygeal approach) and showed good improvement. A coccygeal pillow was advised.

#### 2.1.7. Case 7

A 29-year-old female patient presented with a 6-week coccygeal region (tailbone) pain after falling during cycling. The pain was intense for the first 3 weeks and then improved with NSAID. X-ray showed a type III coccyx (curved sharply and angulated forward). Continued conservative treatment and use of a coccygeal pillow were advised.

Coccydynia refers to pain in and around the coccyx that can arise after major trauma (Case 7) or repetitive minor trauma (Case 6). It is a clinical diagnosis that has no definite correlation with X-ray findings. An impar ganglion block is indicated in coccydynia failing conservative treatment. Cases 6 and 7 emphasized that the clinical response, not the X-ray findings, guides treatment even in the presence of coccyx type IV on X-ray (coccyx fracture or subluxation).

## 3. Discussion

Spine-related pain is a heterogeneous disorder that is commonly correlated with the heterogeneous degenerative and disc disorders. Overemphasizing image findings created a controversy about treatment options and responses which led to (1) undermining conservative treatment in favor of more invasive procedures, (2) variable treatment responses potentially due to undermining the important roles of history and physical examination in identifying pain types and generators, (3) more weight being given to surgical success as seen by images rather than by pain relief and return to functional life, and (4) undermining the research need to develop new investigations that can predict and monitor pain generation and responses like, but not limited to, functional imaging and blood biomarkers. Therefore, degeneration and pain generation mechanisms, as well as pain types and pathways for each generator, are briefly reviewed.

### 3.1. Evolution, Embryogenesis, and Neurulation

Human flexibility, erect posture, and bipedal locomotion expose our spine to increasing degeneration potential. Analysis of the lower lumbar spine from scars in preserved vertebral fossils showed indirect evidence of disc disease in a 1.5-million-year-old* Homo erectus*, whereas Neanderthal spine displayed natural lumbar kyphosis with only mild degeneration [6, 7].

Embryologically, the somites (paraxial mesoderm origin) migrate toward the notochord (origin of nucleus pulposus) forming sclerotomes (origin of the annulus fibrosus and the vertebral bony components), myotomes (future muscles), and dermatomes (future dermis). Myotomes split into epimeres, giving rise to dorsal rami innervated deep intrinsic spine muscles, and hypomeres, giving rise to ventral rami innervated extrinsic spine muscles. The trapezius is derived from the nonsomite lateral mesenchymal plate [8, 9].

Neurulation starts with the formation of the neural plate (future brain and cord) at the level of the fourth somite separated from the surface ectoderm (future epidermis) with neural crest cells (future spinal and cranial nerves sensory ganglia). The neural plate undergoes invagination forming two folds that fuse posteriorly forming the neural tube, with separation of the surface ectoderm and splitting of the neural crest cell layer that migrates from midline to the dorsolateral surface of the neural tube forming dorsal root ganglia (DRG) in the spine. The neural tube grows along its open ends (cranial and caudal neuropores) forming the future brain and spinal cord [10].

### 3.2. Spine Curvature and Flexibility

The critical distinction between human and primate spines is the degree of lordosis and the flexibility of our spine. Bipedalism, long locomotion with hands remaining free in humans, mandated evolutionary adaptations of flexibility, in the human spine curvature; thus creating and maintaining cervical and lumbar lordosis could be considered an active process in comparison with the passively existing thoracic kyphosis. The initiation of cervical and lumbar lordosis is related to the start of head elevation and walking in infancy and childhood. Certain animal breeds have been reported to have disc protrusion, but disc degeneration is unusual in quadrupeds except for those forced to adopt an upright posture like biped rats [11]. Moreover, decreased lumbar lordosis and increased thoracic kyphosis are hallmarks of an aging human spinal column which may indicate returning to the passive natural curvature.

#### 3.2.1. Spine Degeneration and Pain Generators

The main pain generators are the intervertebral disc, facet joint, sacroiliac joint, and spine muscles. Nucleus pulposus of the disc derived from the notochord maintains a unique hypoxic avascular microenvironment with high pressure. Proteoglycans (PG), produced by notochordal cells, play an integral role in maintaining disc function and integrity by imbibing water (acting as sponges) and inhibiting angiogenesis, inflammation, and nerve ingrowth [12].

Disc degeneration describes a cascade of events causing fissures in the annulus fibrosus, water leak, decreasing intradiscal pressure, PG leak leading to inflammation, neovascularization, and ingrowth of free nerve endings to the inner of the disc. Disc innervation occurs mainly via the sinuvertebral nerve from ventral rami and the sympathetic chain, and in a normal disc it penetrates only the outer lamellae [13].

The spine facet is a synovial joint with complex innervation from dorsal rami. The facet joint is formed by two bony articular processes lined with hyaline cartilage and synovial membrane, secreting synovial fluid, forming facet meniscus, and enveloped with a complex fibrous capsule [14]. The facet joint is innervated via medial branches of the dorsal rami where each joint receives a descending branch from the nerve above and an ascending branch from the nerve below (i.e., the L4/5 facet joint is innervated via medial branches from the L3 and L4 dorsal rami).

SIJ pain is an underestimated pain generator with clinical presentation mimicking facet joint and discogenic pain. No specific pain pattern, image findings, or even provocative examination can confirm this diagnosis. Diagnostic injection is the only confirmatory test. Pain mechanisms might be attributed to microinstability caused by weak ligamentous and muscular components which support the effect of periarticular diagnostic injections [15].

Spine muscles contain varying percentages of muscle fiber types I, IIa, and IIb which determine the speed of contraction and fatigue resistance. Postural muscles involved in spine support (deep muscles) showed higher type I fiber, whereas superior trapezius and movement muscles contained fewer type I and more type IIB fibers [16]. Both intrinsic muscles and the strong capsular ligament play a vital role in facet stability via the ligamental-muscular reflex which provides proprioception and organizes the interaction between capsular mechanoreceptors and deep intrinsic muscles.

#### 3.2.2. Pain Types, Pathways, and Possible Generators

Spine pain can be axial, referred, radicular, or a combination of these types.

Axial pain is a nociceptive type that is generated from the disc, facet, sacroiliac joint, muscles, and ligaments from sensitization of the pain receptors (free nerve endings) after tissue injury from mechanical, chemical, or thermal stimuli. Nociceptive spine pain is mediated with sinuvertebral nerves (somatic and autonomic components) from IVD, dorsal rami medial branches from the facet joint, and lateral branches of S1,2,3 from the sacroiliac joint.

Radicular pain is a neuropathic pain type caused by compression and inflammation or injury to a spinal nerve root and/or dorsal root ganglion. This is different from radiculopathy which refers to a complex set of symptoms that can arise from nerve root pathology, including paresthesia, hypoesthesia, anesthesia, and motor loss [17]. The presence of dermatomal distribution in radicular pain is controversial with patients commonly describing the neuropathic radicular pain as deep internal pressure.

Referred pain is a segmental component of nociceptive pain perceived at a location remote from the original injury site. The proposed convergent-projection theory suggests that afferent fibers from different tissues converge into a common second-order neuron in the spinal cord. Therefore, nociception is misinterpreted centrally as originating from other structures [18]. Myofascial pain is a special controversial entity of chronic muscle pain with a hallmark of trigger points and referred pain [2].

A cervicogenic headache is a referred pain to the head or face from soft or bony cervical tissue [16]. Sensory fibers of the trigeminal nerve have their cell bodies in the trigeminal ganglion which is equivalent to the DRG in spinal nerves. Pain and temperature afferents enter the brain stem forming the spinal tract of the trigeminal running caudally to terminate in the spinal nucleus of the trigeminal nerve. Second-order neurons ascend to the contralateral thalamus as trigeminal lemniscus. Upper cervical nerve nociceptive afferents terminate in the spinal trigeminal nucleus sharing common second-order neurons. So, the mechanism of a cervicogenic headache is likely related to convergence between upper cervical and trigeminal nociceptive afferents in the spinal trigeminal nucleus at the upper cervical cord [18, 19]. However, debate exists on pain referral to the trigeminal receptive areas from lower cervical discs.

To summarize complex heterogenous cervical-related pain, four types of pain are generated from the cervical spine: nociceptive, radicular, referred, and trapezius myalgia. Referred pain is a component of nociceptive pain that is perceived in a remote area that shares a common segment or 2nd-order neuron. Axial pain is a nociceptive pain; its referred components, depending on embryological innervation, can be perceived in the skin and are occasionally mistaken with radicular pain, in the muscles or other structures. Radicular pain is a deep, searing, lancinating, or internal pressure that follows the path of the nerve into the arm and may be accompanied by numbness or weakness. Subdivision of radicular symptoms into radicular pain and radiculopathy has been suggested [4]. However, in clinical practice, both terms are used interchangeably. Myofascial pain perceived in the medial side of the scapula is not to be confused with trapezius myalgia. The former is referred pain in the hypomere ventrally innervated muscles (i.e., rhomboids, levator scapulae) that share common innervation with annulus fibrosus, described early in 1959 by Cloward [20], on direct stimulation of the annulus. The latter is a pain in the upper trapezius with debate on the mechanism and neural pathway. Although this is beyond the scope of the current study, it should be mentioned that cervical muscles consist of three layers with different embryological origins, innervation patterns, and possible functions.

Thus, spine pain should not be approached as a single entity even in the same patient. Fragmenting and analyzing pain components with treatment directed to pain generators and mechanisms might give a better response. Image findings are a descriptive diagnosis but not necessarily correlated with patient-presented illness.

The current study has the limitation of a small number of cases; however, the aim was not to compare treatments or outcomes but to emphasize the following from a clinical perspective: the importance of patient history and physical examination, the need for analytical approach, particularly in patients presenting with multiple pain patterns and types, the avoidance of overestimating image findings, and the research needs for functional imaging and biomarkers.

## 4. Conclusion

Spondylosis is the asymptomatic process of spine degeneration detected primarily with imaging. The clinical presentation is related to stenosis or pain. The mechanism of pain might be related to one or more combinations of compression, inflammation, or microinstability. Two major pain types occur in the spine: nociceptive as in axial pain and neuropathic as in radicular pain from disc herniation. Referred pain is a component of nociceptive pain. Pain generators (IVD, facet, SIJ, muscles, and ligaments) might coexist in the same patient causing mixed pain types and referral patterns with multiple mechanisms and pathways. Patient history, physical examination, and diagnostic injections are the mainstay for diagnosis and treatment, with imaging as an assisting but occasionally confusing factor. Hence, there is a need for new functional imaging and biomarkers.

## Data Availability

Data used to support the findings of this study are available from the corresponding author upon request.
